# Comparing the effectiveness of efavirenz and nevirapine for first-line antiretroviral treatment amongst an adult treatment cohort from South Africa

**DOI:** 10.1186/1758-2652-13-S4-P10

**Published:** 2010-11-08

**Authors:** P Bock, G Fatti, A Grimwood

**Affiliations:** 1 Kheth Impilo, University of Cape Town, Cape Town, South Africa

## Purpose of the study

There is an ongoing debate about when to use efavirenz (EFV) or nevirapine (NVP) for first line antiretroviral treatment (ART) in developing countries, fuelled by EFV's link with teratogenicity in rats and NVP's risk of hepatotoxicity with increasing CD4 levels and it's interactions with Rifampicin. This paper compares the effectiveness of these two drugs in a multicentre adult cohort of ART patients attending public health facilities in South Africa.

## Methods

A retrospective cohort analysis of routine data on 27350 ART naïve adults initiated between March 2004 and March 2007 at 56 public health sites across 4 provinces was completed. Stata 9 was used to conduct analyses which included Kaplan Meir survival analysis, logistic regression, generalised estimating equations and Cox proportional hazard models.

## Summary of results

Median follow up time was 9.3 months and median baseline CD4 count 113 cells/mL (IQR= 57-165). Multivariate analyses showed patients receiving first line regimens containing EFV to have been more likely to suppress virologically both at 6 months (OR=1.30; 95%CI:1.11-1.54) and at any time between 6 and 36 months (OR= 1.28; 95%CI:1.16-1.41), less likely to change regimen (OR=0.53; 95%CI:0.48-0.59) and more likely to die (AHR= 1.24; 95%CI:1.07-1.45). A subset analysis of 18527 patients with pregnancy and tuberculosis status reported showed no difference in hazard rates for death between the two groups (AHR=1.17; 95%CI:0.99-1.37).

## Conclusions

This data shows superior results for patients on EFV with respect to all outcomes except death. Retrieval of missing data about pregnancy and tuberculosis status may push this last association toward the null. Protease inhibitors are an alternative to non-nucleoside reverse transcriptase inhibitors (NNRTIs) for first line use; but are currently too costly. There is an urgent need for further research into currently available NNRTIs; as well as the development of new antiretrovirals for resource depleted settings. In the interim the developing world needs to increase access and bring down the cost of existing drugs and implement more efficient treatment strategies.

